# Early Warning Scores in Emergency Department Patients Aged 80 Years or Older

**DOI:** 10.1001/jamanetworkopen.2026.1532

**Published:** 2026-03-19

**Authors:** Marcello Covino, Piergiacomo Maria Cacciamani Fanelli, Nicola Bonadia, Valeria Maccauro, Davide Antonio Della Polla, Giuseppe De Matteis, Andrea Piccioni, Antonio Gasbarrini, Claudio Sandroni, Francesco Franceschi

**Affiliations:** 1 Emergency Department, Fondazione Policlinico Universitario A. Gemelli, IRCCS, Rome, Italy; 2 Università Cattolica del Sacro Cuore, Rome, Italy; 3 Department of Geriatrics, Fondazione Policlinico Universitario A. Gemelli, IRCCS, Rome, Italy; 4 Department of Internal Medicine and Gastroenterology, Fondazione Policlinico Universitario A. Gemelli, Rome, Italy; 5 Department of Anesthesiology and Intensive Care Medicine, Fondazione Policlinico Universitario A. Gemelli, IRCCS, Rome, Italy

## Abstract

**Importance:**

Early warning scores (EWSs) are widely used tools to support triage and risk stratification in the emergency department (ED). However, data on their performance in identifying clinical deterioration among acutely ill adults aged 80 years or older are scarce.

**Objective:**

To evaluate and compare the performance of 5 EWSs for predicting short-term clinical deterioration in ED patients aged 80 years or older.

**Design, Setting, and Participants:**

This retrospective prognostic study included all consecutive nontrauma ED visits by patients aged 80 years or older between January 2015 and December 2024 at a large urban teaching hospital in Rome, Italy.

**Exposures:**

Physiologic parameters recorded at ED admission were used to calculate 5 EWSs: National Early Warning Score (NEWS), National Early Warning Score 2 (NEWS2), Modified Early Warning Score (MEWS), Rapid Emergency Medicine Score (REMS), and International Early Warning Score (IEWS).

**Main Outcomes and Measures:**

The primary outcome was clinical deterioration, defined as death or intensive care unit (ICU) admission within 24 hours of ED arrival. Discrimination was assessed by area under the receiver operating characteristic curve (AUROC), calibration by the Brier score, and age-related performance by spline regression. Comparative contributions of variables were analyzed using Shapley additive explanations (SHAP) values.

**Results:**

Among 50 645 patients (median age, 85 years [IQR, 82-88 years]; 54.6% females), 1233 (2.4%) experienced clinical deterioration. All EWSs demonstrated fair discrimination (AUROC range, 0.747 [IQR, 0.731-0.763] for MEWS to 0.782 [IQR, 0.767-0.798] for NEWS). Whereas NEWS achieved the highest AUROC, REMS had the best calibration (Brier score, 0.0220; 95% CI, 0.0208-0.0232). Discriminatory performance declined with increasing age beyond 90 years except for REMS, whose predictive accuracy improved among patients older than 94 years. For patients aged 87 years or older vs 80 to 86 years, oxygen supplementation (SHAP difference, 0.59), systolic blood pressure (SHAP difference, 0.32), and Glasgow Coma Scale score (SHAP difference, 0.40) were the strongest predictors of clinical deterioration.

**Conclusions and Relevance:**

In this prognostic study of EWSs conducted among ED patients aged 80 years or older, all scores provided acceptable short-term prognostic accuracy. REMS demonstrated the most consistent performance in patients aged 94 years or older, supporting its use for targeted risk stratification in this population.

## Key Points

**Question:**

Do early warning scores (EWSs) accurately predict short-term clinical deterioration among older patients in the emergency department (ED)?

**Findings:**

In this prognostic study including 50 645 Italian patients aged 80 years or older, 5 evaluated EWSs showed fair discrimination. The National Early Warning Score (NEWS) achieved the highest area under the curve, while the Rapid Emergency Medicine Score (REMS) showed superior calibration and positive predictive value and was the only score whose performance improved with increasing age.

**Meaning:**

NEWS and its derivatives offered greater sensitivity, whereas REMS provided higher precision that may reduce alarm fatigue and outperformed other scores in older patients.

## Teaser

This prognostic study assesses the accuracy of 5 early warning scores to predict death or intensive care unit admission within 24 hours of being seen in the emergency department in patients aged 80 years or older in Italy.

## Introduction

The worldwide increase in life expectancy has led to a growing number of older adults accessing emergency departments (EDs). In the US, patients aged 65 years or older accounted for approximately 20% of all ED visits, totaling 33 million, in 2022.^1^ Similar trends are observed in Europe, where adults aged 65 years or older represent the fastest-growing ED patient population.^2^

This demographic shift poses significant challenges for health care systems. Older patients often present with complex medical conditions and multiple comorbidities, increasing the risk of adverse outcomes. Additionally, factors such as cognitive impairment, functional decline, reduced physiologic reserve, and atypical symptom presentation complicate diagnosis and management in the fast-paced ED environment.^3,4,5,6^

Early and accurate identification of high-risk patients is essential to optimize diagnostic and therapeutic strategies and improve outcomes. Early warning scores (EWSs) are widely used tools that quantify physiologic parameters to detect clinical deterioration. Initially developed for hospitalized patients, EWSs have been adapted for ED use to support triage and risk stratification.^7,8^

However, the predictive performance of EWSs in adults aged 65 years or older may be suboptimal.^9^ Blunted physiologic responses can result in deceptively low scores despite severe illness, while minor deviations from baseline may yield high scores, potentially leading to alarm fatigue and increased clinical workload without improving outcomes.^10,11,12,13^

Although EWSs are widely used to identify clinical deterioration in acutely ill patients and have been studied in populations aged 65 years or older, data on their performance in those aged 80 or more years remain scarce. This study aimed to evaluate the predictive accuracy of the most widely used EWSs for short-term clinical deterioration in a large cohort of ED patients aged 80 years or older.

## Methods

### Study Design and Population

This single-center prognostic study was conducted in the ED of an urban teaching hospital in Rome, Italy, serving a catchment area of approximately 1.8 million inhabitants, with a mean of about 75 000 ED visits per year. We retrospectively reviewed electronic medical records of all consecutive patients aged 80 years or older admitted to the ED for nontraumatic conditions between January 2015 and December 2024. This study was approved by the Local Ethics Committee of Lazio—Area 3 and was performed following the ethical standards established in the 1964 Declaration of Helsinki and its later amendments.^16^ Since the patients’ data were anonymized, informed consent was waived by the ethics committee. The study was classified as type 4 according to and was conducted following the Transparent Reporting of a Multivariable Prediction Model for Individual Prognosis or Diagnosis (TRIPOD)^14^ reporting guideline.^15^

### Exclusion Criteria

Patients were excluded if they were seen for trauma; were intubated or required cardiopulmonary resuscitation by the prehospital emergency service prior to ED arrival; were in cardiocirculatory arrest at ED arrival; or died, experienced cardiac arrest, were intubated, or were admitted to a general or specialty intensive care unit (ICU) within 1 hour of being seen in the ED. Patients with more than 2 missing parameters required for EWS calculation were also excluded.

### Data Collection

Investigators blinded to patient outcomes extracted the following data from electronic records: age, sex, clinical presentation, temperature, heart rate, respiratory rate, blood pressure, Glasgow Coma Scale (GCS) score, oxygen supplementation, peripheral oxygen saturation (SpO_2_), radiographic imaging, and clinical history. The physiologic values used for EWS calculation were those recorded at ED admission.

For patients with 1 or 2 missing parameters, data were imputed using a multiple imputation approach.^17^ Imputed values were incorporated into the original dataset to generate complete vital sign profiles for EWS computation. Details of the imputation process are provided in the eMethods in Supplement 1.

### Score Calculation

The following EWSs were calculated for each patient: National Early Warning Score (NEWS),^18,19^ National Early Warning Score 2 (NEWS2),^20,21^ Modified Early Warning Score (MEWS),^22^ Rapid Emergency Medicine Score (REMS),^23^ and International Early Warning Score (IEWS).^24^ For NEWS2, patients were considered at risk of type 2 respiratory failure if they had a confirmed history of chronic obstructive pulmonary disease.

### Study End Points

The primary end point was clinical deterioration, defined as death or ICU admission within 24 hours of ED arrival. Secondary end points included (1) analysis of the comparative contribution of individual score components to deterioration prediction and (2) evaluation of how these contributions varied with increasing age.

### ICU Admission Criteria

ICU admission criteria remained consistent throughout the study period. Indications included the need for advanced respiratory support or severe shock requiring inotropic therapy and invasive monitoring.

### Statistical Analysis

Continuous variables are reported as medians (IQRs) and were compared using the Mann-Whitney *U* test. Categorical variables are presented as absolute numbers (percentages) and were compared using the χ^2^ test or Fisher exact test, as appropriate. For patients with 1 or 2 missing parameters, imputation was performed using Monte Carlo methods; all imputed variables had less than 5% missing data points. Score performance was assessed in terms of discrimination and calibration. Discrimination refers to the score’s ability to distinguish between patients with and without the outcome, while calibration reflects the agreement between predicted and observed event probabilities.

Receiver operating characteristic (ROC) curve analysis was used to evaluate discrimination. Areas under the ROC curve (AUROC) were compared using the DeLong method.

Calibration was assessed using Brier scores, with 95% CIs and *P* values derived from bootstrap resampling, and results were compared with both a null model and the best-performing score. Additionally, calibration plots illustrating observed vs predicted probabilities were generated for each EWS, with calibration slopes derived from logistic regression models including each EWS as the sole predictor.

The discriminative performance of EWSs was evaluated across patient age using a spline-based, age-stratified analysis. Sliding age windows of 5 years, advancing in 1-year increments, were applied to compute the AUROC within each interval. The association between age and AUROC was modeled with restricted cubic splines fitted via ordinary least squares regression with 3 degrees of freedom. Spline regression models were compared with fixed-age (intercept-only) models to determine the statistical significance (*F* test) of age effects.

An extreme gradient boosting (XGBoost) model incorporating core variables from all scores was developed to assess the comparative contribution of individual score components. Contributions were quantified using raw and normalized (divided by the maximum value) Shapley additive explanations (SHAP) values. Separate XGBoost analyses, consistent with the previously described modeling framework, were performed to assess differences in the comparative contributions of predictors within each age stratum. All modeling procedures, including data preprocessing and hyperparameter settings, were identical across groups to maintain methodologic consistency. Differences in SHAP values between age groups were evaluated using 2-sided nonparametric bootstrap tests on the difference in normalized absolute SHAP values.

Data were analyzed using IBM SPSS for Windows, version 25 (IBM Corp); MedCalc, version 19.2.1 (MedCalc Ltd); and R, version 4.5.1 (R Project for Statistical Computing).^25^ Custom programming in Python, version 3.13 (Python Software Foundation), was used to run XGBoost analysis and calculate SHAP values. Two-sided *P* ≤ .05 was considered statistically significant. A comprehensive description of the analytic methods is available in the eMethods in Supplement 1.

## Results

**Figure 1.**
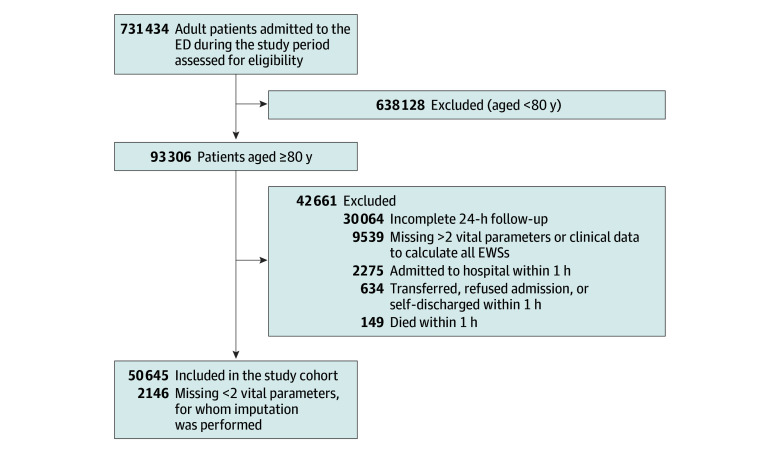
Flow Diagram Summarizing the Inclusion Process ED indicates emergency department; EWS, early warning score.

Overall, 50 645 patients aged 80 years or older were included in the study. The median age was 85 (IQR, 82-88) years; 27 644 patients (54.6%) were females and 23 001 (45.4%) were males. A clinical deterioration occurred in 1233 patients (2.4%). The inclusion process is summarized in Figure 1, while a summary of physiologic and clinical parameters in the study cohort is presented in the Table and the distribution of different scores is represented in eFigure 1 in Supplement 1.

**Table.** Summary Statistics of Physiologic and Demographic Variables

Variable	Patient group^a^		
	All (n = 50 645)	Controls (n = 49 412)	Died or admitted to ICU within 24 h (n = 1233)
Age, median (IQR), y	85 (82-88)	85 (82-88)	85 (82-89)
Sex			
Female	27 644 (54.6)	26 969 (54.6)	675 (54.7)
Male	23 001 (45.4)	22 443 (45.4)	558 (45.3)
Vital parameters			
Heart rate, bpm	80 (69-90)	80 (69-90)	86 (100-107)
Respiratory rate, breaths/min	19 (16-23)	19 (16-23)	20 (17-23)
MAP, mm Hg	97 (84-108)	97 (85-108)	85 (70-103)
Blood pressure, mm Hg			
Systolic	139 (120-158)	139 (120-158)	120 (93-143)
Diastolic	75 (65-85)	75 (65-86)	70 (55-81)
Sao_2_	96 (94-98)	96 (94-98)	92 (85-96)
Body temperature, °C	36.5 (36.0-37.1)	36.5 (36.0-37.1)	36.5 (36.0-37.3)
GCS score^b^	15 (14-15)	15 (14-15)	14 (11-15)
Supplementary O_2_	4756 (9.4)	4339 (8.8)	417 (33.8)
T2RF	3297 (6.5)	3162 (6.4)	135 (11.00)
Clinical symptoms			
Not alert (AVPU = U)	3140 (6.2)	2708 (5.5)	432 (35.0)
Dyspnea	9117 (18.0)	8638 (17.5)	479 (38.8)
Chest pain	3839 (7.6)	3792 (7.7)	47 (3.8)
Abdominal pain	5488 (10.8)	5396 (10.9)	92 (7.5)
Syncope	3784 (7.5)	3724 (7.5)	60 (4.9)
Vertigo	1203 (2.4)	1201 (2.4)	2 (0.2)
Oliguria	1197 (2.4)	1149 (2.3)	48 (3.9)
Fever	6894 (13.6)	6708 (13.6)	186 (15.1)
Diarrhea	1614 (3.2)	1572 (3.2)	42 (3.4)
Emesis	3805 (7.5)	3697 (7.5)	108 (8.8)
EWS			
NEWS	2 (0-2)	2 (0-4)	6 (3-8)
NEWS2	2 (0-4)	2 (0-4)	5 (3-8)
MEWS	1 (0-2)	1 (0-2)	3 (2-4)
REMS	6 (6-8)	6 (6-8)	9 (8-11)
IEWS	8 (7-11)	8 (7-11)	12 (10-15)

Abbreviations: AVPU, Alert, Verbal, Pain, and Unresponsive scale; bpm, beats per minute; EWS, early warning score; GCS, Glasgow Coma Scale; ICU, intensive care unit; IEWS, International Early Warning Score; MAP, mean arterial pressure; MEWS, Modified Early Warning Score; NEWS, National Early Warning Score; NEWS2, National Early Warning Score 2; O_2_, oxygen; REMS, Rapid Emergency Medicine Score; Sao_2_, arterial oxygen saturation; T2RF, type 2 respiratory failure; U, unresponsive.

^a^ Metric variables and EWSs are reported as median (IQR); nominal and ordinal values are reported as No. (%) of patients.

^b^ Score range, 3 to 15, with lower scores indicating greater impairment of consciousness.

### Score Discrimination Performance

All the evaluated EWSs had a fair discrimination performance. The median AUROCs were 0.782 (IQR, 0.767-0.798) for NEWS, 0.776 (IQR, 0.761-0.791) for NEWS2, 0.752 (IQR, 0.736-0.768) for REMS, 0.747 (IQR, 0.731-0.763) for MEWS, and 0.779 (IQR, 0.764-0.795) for IEWS. Compared with the best predictor (NEWS), all the evaluated EWSs had a slightly though statistically significantly lower AUROC (eFigure 2 and eTable 1 in Supplement 1). Despite exhibiting strong overall discriminatory power, EWSs demonstrated limited positive predictive value (PPV), primarily due to the small number of observed adverse events (eTables 2 and 3 in Supplement 1). REMS and MEWS achieved PPVs closer to optimal values across varying thresholds than the other 3 EWSs.

### Score Calibration

**Figure 2.**
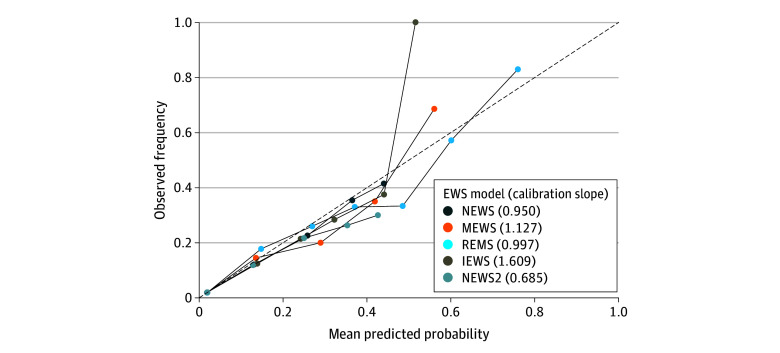
Calibration Plot of Predicted Probabilities and Observed Outcome Frequencies for 5 Early Warning Score (EWS) Logistic Regression Models Dashed diagonal line represents perfect calibration (slope = 1), where predicted risk matches observed risk. Models with a slope less than 1 indicate overfitting, where predictions are too extreme, and models with a slope greater than 1 indicate underfitting, where predictions are too conservative. IEWS indicates International Early Warning Score; MEWS, Modified Early Warning Score; NEWS, National Early Warning Score; NEWS2, National Early Warning Score 2; REMS, Rapid Emergency Medicine Score.

Based on the Brier score analysis, REMS was the most well-calibrated EWS within the study population (Brier score, 0.0220; 95% CI, 0.0208-0.0232), with significantly superior performance compared with NEWS, NEWS2 (highest Brier score at 0.0229; 95% CI, 0.0216-0.0241), IEWS, and MEWS. The Brier score of the null model was 0.0238 (95% CI, 0.0225-0.0250). Evaluation of calibration slopes across varying threshold values further confirmed REMS as the top performer, with a slope of 0.997, followed by NEWS with a slope of 0.950 (Figure 2 and eTable 4 in Supplement 1).

### Classification

**Figure 3.**
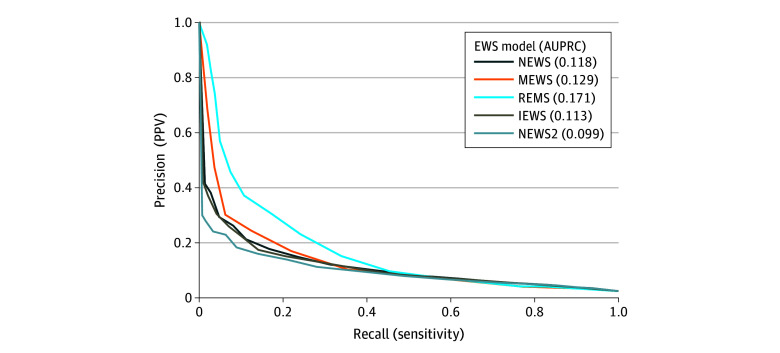
Precision-Recall Curves Comparing Early Warning Scores (EWSs) AUPRC indicates area under the precision-recall curve; IEWS, International Early Warning Score; MEWS, Modified Early Warning Score; NEWS, National Early Warning Score; NEWS2, National Early Warning Score 2; PPV, positive predictive value; REMS, Rapid Emergency Medicine Score.

Classification performance metrics are reported in eTables 2 and 3 and eFigure 3 in Supplement 1. Precision-recall curves for the different EWSs are shown in Figure 3. Overall, all tested scores showed low to moderate sensitivity, with a marked decline at the 10% PPV threshold.

At the lowest 5% margin, IEWS showed the highest sensitivity (78.1%; 95% CI, 75.7%-80.3%), closely followed by NEWS (71.9%; 95% CI, 69.4%-74.4%) and NEWS2 (71.2%; 95% CI, 68.6%-73.7%), whereas REMS (57.7%; 95% CI, 54.9%-60.4%) and MEWS (54.1%; 95% CI, 51.3%-56.9%) showed lower sensitivity. Sensitivity declined sharply at higher PPV margins: at the 10% PPV threshold, NEWS showed the highest sensitivity (41.5%; 95% CI, 38.8%-44.3%), followed by IEWS (37.5%; 95% CI, 34.8%-40.2%), MEWS (35.4%; 95% CI, 32.8%-38.2%), REMS (33.9%; 95% CI, 31.3%-36.6%), and NEWS2 (28.1%; 95% CI, 25.6%-30.6%).

In contrast, specificity was higher at increasing PPV thresholds. At the 5% PPV threshold, the highest specificity was observed for MEWS (83.5%; 95% CI, 83.1%-83.8%), followed by REMS (81.4%; 95% CI, 81.0%-81.7%), NEWS (70.1%; 95% CI, 69.7%-70.5%), NEWS2 (69.6%; 95% CI, 69.2%-70.0%), and IEWS (63.5%; 95% CI, 63.1%-63.9%). At the 10% threshold, REMS had the highest specificity (95.3%; 95% CI, 95.1%-95.5%), followed by NEWS2 (94.5%; 95% CI, 94.3%-94.7%), MEWS (92.3%; 95% CI, 92.1%-92.5%), IEWS (92.2%; 95% CI, 91.9%-92.4%), and NEWS (90.8%; 95% CI, 90.5%-91.0%). Owing to the low event rate, negative predictive value (NPV) remained high at each threshold for all the scores. At the lowest 5% threshold, the highest NPV was observed for IEWS (99.1%; 95% CI, 99.0%-99.2%), followed by both NEWS and NEWS2 (99.0%; 95% CI, 98.9%-99.1%), REMS (98.7%; 95% CI, 98.6%-98.8%), and MEWS (98.6%; 95% CI, 98.5%-98.8%). At the 10% margin, the highest NPV was achieved by NEWS (98.4%; 95% CI, 98.3%-98.5%), followed by MEWS, REMS, and IEWS (all 98.3%; 95% CI, 98.2%-98.4%) and lastly by NEWS2 (98.1%; 95% CI, 98.05%-98.3%). Conversely, MEWS and REMS yielded the highest PPVs across all risk thresholds. Specifically, at the lowest 5% margin, MEWS showed the highest PPV (7.6%; 95% CI, 7.0%-8.1%), followed by REMS (7.2%; 95% CI, 6.7%-7.7%), NEWS (5.7%; 95% CI, 5.3%-6.0%), NEWS2 (5.5%; 95% CI, 5.2%-5.9%), and IEWS (5.1%; 95% CI, 4.8%-5.4%). At the 10% margin, the highest PPV was achieved by REMS (15.2%; 95% CI, 13.9%-16.6%), followed by NEWS2 (11.3%; 95% CI, 10.2%-12.4%), IEWS (10.7%; 95% CI, 9.8%-11.6%), MEWS (10.3%; 95% CI, 9.4%-11.2%), and NEWS (10.1%; 95% CI, 9.3%-11.0%).

### Score Performance According to Age

**Figure 4.**
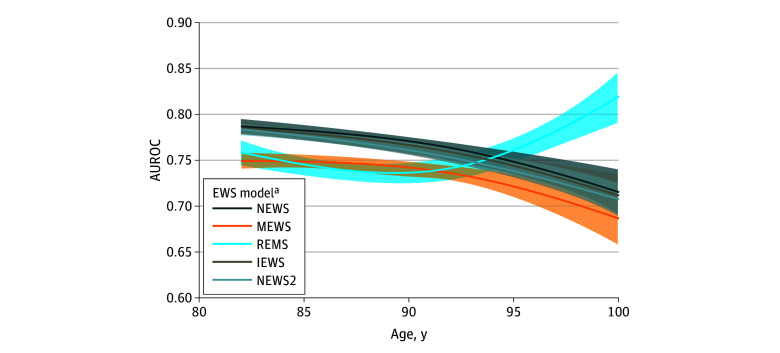
Spline Curve Analysis of Area Under the Receiver Operating Characteristic Curve (AUROC) by Age for Early Warning Score (EWS) Models *P* values from the spline regressions indicate an association between age and AUROC. Shaded areas represent the 95% CIs in the AUROC of each EWS score. IEWS indicates International Early Warning Score; MEWS, Modified Early Warning Score; NEWS, National Early Warning Score; NEWS2, National Early Warning Score 2; REMS, Rapid Emergency Medicine Score. ^a^All *P* < .001.

Spline curve analysis revealed a significant association between age and the performance of EWSs. NEWS, NEWS2, MEWS, and IEWS exhibited a decline in discriminatory capacity with advancing age, with the most pronounced reduction observed in individuals aged more than 90 years. In contrast, REMS—despite demonstrating comparatively lower overall discrimination performance—showed an age-associated improvement, ultimately surpassing the other EWSs in predictive accuracy among patients older than 94 years (Figure 4).

### Comparative Contribution of Each Item to Deterioration Prediction

To identify the factors associated with score performance in older patients, we compared the 32 723 patients (64.6%) aged 80 to 86 years with the 17 922 patients (35.4%) aged 87 years or older. SHAP value analysis revealed that except for peripheral oxygen saturation, all EWS components exhibited different contributions in patients aged 87 years or older compared with those aged 80 to 86 years (eFigures 4 and 5 in Supplement 1). When assessing the comparative importance of each variable in predicting adverse outcomes among patients aged 87 years or older vs those aged 80 to 86 years, the most pronounced differences were observed for oxygen supplementation (SHAP difference, 0.59), GCS score (SHAP difference, 0.40), AVPU (Alert, Verbal, Pain, and Unresponsive) scale (SHAP difference, –0.38), and systolic blood pressure (SHAP difference, 0.32) (eTables 5 and 6 and eFigures 4 and 5 in Supplement 1).

## Discussion

EWSs emerged in the late 1990s^26^ after evidence that many in-hospital deaths were preceded by long periods of clinical deterioration, allowing time for therapeutic escalation.^27,28,29^ More recently, machine learning algorithms have been introduced to enhance predictive performance, resulting in the emergence of proprietary, closed-source models. These developments have progressively blurred the distinction between traditional physiologic scoring systems and outcome prediction tools.^30^

However, the derivation and validation cohorts for most available scores included patients across a wide age spectrum, and although they generally incorporated a substantial proportion of older individuals, the median ages ranged from 50 to 72 years.^31^ A previously published review identified 34 scores developed for unselected medical patients, 70% of which were data driven; however, most derivation and validation studies failed to meet established reporting criteria.^31^ Concerns have also been raised regarding potential inflation of reported accuracy.^32^

As a consequence of demographic transition, older patients now represent an increasingly significant share of health care resource utilization.^1,2^ This population also differs substantially from the cohorts on which most EWSs were originally developed. Consistent with this observation, the inclusion of age has been suggested as a means to improve EWS performance.^33^

To address this gap, we performed a retrospective comparison of established EWSs in patients aged 80 years or older admitted to the ED. We evaluated model performance in terms of classification, discrimination, and calibration. To our knowledge, this is the largest ED-based validation of EWSs in this age group to date.

Overall, all the scores we tested showed a fair to good discriminative capacity in the study population (range of median AUROCs, 0.747-0.782). While a modest reduction was observed compared with original derivations,^18,19,20,21,22,23,24^ it likely reflects the known limitation of physiologic scoring systems in geriatric populations. Despite not including age, the NEWS showed the highest AUROC among our scores, higher than the IEWS, which was specifically developed to include age and sex. The MEWS and the REMS, conversely, showed the lowest and second lowest AUROCs, respectively. However, the overall difference between the highest and the lowest AUROC value was only 0.035 units (calculated at the point estimate). These results align with previous literature where AUROC values between 0.768 and 0.94 for short-term mortality were reported.^34^ However, more recent studies specifically focused on the geriatric population have reported heterogenous and sometimes conflicting results. For example, a recent prospective study including more than 3000 patients older than 65 years found that the Triage Early Warning Score outperformed NEWS, REMS, and MEWS in predicting mortality at different time points, as well as ICU admission.^35^ Conversely, a multicenter study comparing the discriminative performance of EWSs in adults aged 18 to 64 years vs 65 years or older reported a reduced discriminatory capability of traditional EWSs in the older group.^10^

The evaluation of PPV—which reflects the likelihood of a true adverse event when a patient is classified as high risk—revealed a performance hierarchy distinct from that suggested by ROC-derived metrics (eFigure 2 in Supplement 1). REMS and MEWS achieved PPVs closer to optimal values across varying thresholds, outperforming NEWS, NEWS2, and IEWS in this regard. This discrepancy is partly attributable to the distributional characteristics of the latter scores, which tend to cluster a substantial proportion of patients within the moderate-risk range. It may also reflect the different aims underlying the scores’ development: the NEWS family was developed to guide the frequency of patient reassessment in general wards, where a more granular distinction across different risk categories is desirable, whereas REMS was specifically developed for EDs, with the explicit purpose of guiding care escalation in acutely ill patients. As a result, when clinical thresholds are applied to identify high-risk individuals, the elevated sensitivity of NEWS and its derivatives at moderate-risk levels leads to an increase in false positives. This, in turn, reduces PPV and undermines the reliability of high-risk classifications necessary for targeted clinical interventions. The discrepancy between AUROC and PPV thus illustrates a possible trade-off between sensitivity and low-yield alarm rate: scores optimized for their statistical accuracy might not translate into higher operational effectiveness than slightly less precise scores.^36^ This is particularly the case in an ED, where rapid triage and finite response resources matter, so that higher PPV can be more actionable than marginal AUC gains.

Calibration analysis using the Brier score showed that REMS had the lowest value among all scores (score range, 0.0220-0.0229), indicating better alignment with observed outcomes. These differences were modest, reflecting the low event rate and the low baseline Brier score of the null classifier (0.0238). Calibration remains a critical aspect of clinical decision-making.^37^

Our findings highlight the complex interplay between advanced age and the predictive reliability of conventional EWS models. Through spline curve analysis, we identified a significant association of age with EWS performance, with a general decline in discriminatory capacity observed for NEWS, NEWS2, MEWS, and IEMS as patient age increased. This reduction was most pronounced among individuals aged 90 years or older. Notably, REMS exhibited an exception to this trend, demonstrating an age-associated improvement in predictive accuracy that enabled it to outperform other EWS models in patients aged over 94 years. Compared with IEWS, this is somewhat surprising, since REMS points for age flatten at 74 years, while the IEWS increases more gradually.^21^ This is consistent with the idea that in ED patients, higher physiologic derangement may correlate better with early outcomes than baseline age-related fragility.

The comparative contribution of individual scoring items explored by an XGBoost model revealed that among patients aged 87 years or older, the variables with the greatest average contribution to outcomes were oxygen supplementation, SpO_2_, systolic blood pressure, GCS score, and heart rate. In this age group compared with patients aged 80 to 86 years, the influence of oxygen supplementation, GCS score, and systolic blood pressure increased significantly. These findings suggest that in patients aged 87 years or older, the association between physiologic derangement and clinical deterioration becomes more pronounced and that the GCS score, which is able to capture early neurologic deterioration, shows a better correlation with the overall risk than does AVPU. This pattern aligns with our main findings: the REMS score, which applies a less aggressive age penalty than IEWS and offers finer stratification of vital sign abnormalities than NEWS or NEWS2, was the most discriminative tool among patients aged 94 years or older.

In addition, all EWSs are developed using routinely collected clinical data, thus capturing not only biological associations but also patterns of clinical behavior and decision-making. The association between physiologic derangement and outcomes in patients aged 87 years or older may therefore reflect both increased biological vulnerability and differences in clinical management intensity or expectations of recovery.

A key finding of this study is that all tested scores performed well in the population aged 80 years or older. This constitutes a geographic, temporal, and cross-domain validation. Notably, all the included scores rely on predictors grounded in established clinical knowledge. This observation bridges performance evaluation of EWSs into the broader discourse on model interpretability. From this perspective, interpretability is not merely a practical advantage; instead, it reflects the alignment between prediction models and known biological mechanisms, serving as a proxy for epistemic robustness. In bayesian terms, predictors based on mechanistic understanding and repeated clinical observation are more likely to capture genuine biological processes than those derived from automated feature selection. Cross-domain consistency may therefore stem from this adherence to biological plausibility, whereas data-driven models risk overfitting data while underfitting reality.^38^

A well-known example is a study by Zech et al^39^ in which a neural network identified the word *portable* as a predictor of pneumonia in chest radiography reports—correctly associating it with institutional practices rather than pathology. This issue is particularly relevant in the context of closed-source models, where commercial incentives may prioritize apparent accuracy and the lack of transparency hinders independent validation.

Indeed, recent evidence suggests that outperforming clinically derived scores requires large datasets and substantial computational resources, yet the most influential predictors remain unchanged. A validation study demonstrated that eCART—a machine learning–based, transparent, and interpretable score^40^—achieved clinically relevant gains over NEWS.^41^ However, these gains were obtained by incorporating 97 predictors, including multiple trends and documentation features, with a gradient-boosted model trained on millions of observations. Notably, despite this increased complexity, the most influential predictors largely overlapped with those included in NEWS, and proprietary closed-source algorithms performed worse than both eCART and NEWS in the same study. Another example is the Advanced Alert Monitor (AAM),^42^ among the few predictive models for which patient-centered outcome data are available.^43^ Importantly, AAM required substantial computing power and a dedicated organizational infrastructure, raising questions about its cost-effectiveness and scalability.

### Limitations

The main limitation of this study is its single-center, retrospective design. It was conducted in a large urban teaching hospital, which may limit generalizability. Additionally, the comparison between scores was restricted to their ability to predict clinical deterioration within the first 24 hours of ED arrival. While consistent with the ED focus, this may overlook relevant deterioration occurring beyond this window. Our results may also be influenced by our choice to exclude patients who died, were admitted to an ICU-level unit, or required intubation or cardiopulmonary resuscitation in the first hour since ED arrival. Those patients were excluded because EWSs are unlikely to provide additional prognostic or decision-making value when a critical condition is already apparent upon arrival or in patients whose condition mandates early ICU admission according to institutional protocols (eg, ST-segment elevation myocardial infarction or acute stroke). While not an established standard, the 1-hour time frame was chosen as a pragmatic proxy to identify this subgroup of patients. Also, each patient encounter was considered a single observation, and no correction was made for early readmissions by the same patient. This approach may introduce dependency between observations and limit generalizability. However, given the large catchment area and the high volume of ED visits, this limitation is unlikely to have a meaningful impact on the overall results. In addition, given the possible similarity between our institution and those where the scores were originally developed or validated, our findings may reflect reproducibility rather than broader generalizability.^44^

## Conclusions

In this prognostic study, all evaluated EWSs demonstrated satisfactory performance in predicting short-term clinical deterioration among patients aged 80 years or older seen in the ED. The REMS showed superior accuracy in the subgroup aged more than 94 years and was particularly effective in identifying a smaller cohort of high-risk patients, albeit with reduced sensitivity. Conversely, the NEWS and NEWS2 offered slightly higher sensitivity but appeared more susceptible to alarm fatigue. Therefore, the choice of a specific scoring system should be guided by local operational requirements and clinical context. However, given their inherent limitations and potential biases, EWSs should be regarded as supportive tools rather than substitutes for clinical judgment in the population older than 80 years.

## Notes

Supplement 1.
eMethods
**eTable 1.** Discriminatory Ability of Early Warning Scores in Predicting Death or ICU Admission Within 24 Hours From ED Arrival **eTable 2.** Performance of EWSs for Predicting Death or ICU Admission Within 24 Hours From Emergency Department Arrival **eTable 3.** Performance of EWSs for Predicting Death or ICU Admission Within 24 Hours From Emergency Department Arrival at Discrete PPV Thresholds **eTable 4.** Calibration Accuracy of EWSs Based on Brier Scores for Predicting 24-Hour Death or ICU Admission Upon Emergency Department Arrival **eTable 5.** Predictor Importance for Extreme Gradient Boosting Model Predicting Unexpected Death or ICU Admission Within 24 Hours of Emergency Department Arrival in Patients Aged 80 or More Years **eTable 6.** Predictor Importance for 2 Separate Extreme Gradient Boosting Models Predicting Unexpected Death or ICU Admission Within 24 Hours of Emergency Department Arrival **eFigure 1.** Violin Plots Showing the Distribution of Risk Score Values Stratified by 24-Hour Outcome **eFigure 2.** ROC Curve Analysis of the Evaluated EWS **eFigure 3.** Positive Predictive Value Across EWS Thresholds **eFigure 4.** SHAP Values **eFigure 5.** SHAP Summary Plots for Extreme Gradient Boosting Models Predicting Unexpected Death or ICU Admission Within 24 Hours of Emergency Department Arrival in 2 Age Groups

Supplement 2.
Data Sharing Statement
