# Supplementary material for: Early Warning Scores in Emergency Department Patients Aged 80 Years or Older
Source: JAMA Netw Open. 2026 Mar 19;9(3):e261532. doi: 10.1001/jamanetworkopen.2026.1532 (PMC13003372; doi:10.1001/jamanetworkopen.2026.1532)
Supplement: Supplement 1. — eMethods eTable 1. Discriminatory Ability of Early Warning Scores in Predicting Death or ICU Admission Within 24 Hours From ED Arrival eTable 2. Performance of EWSs for Predicting Death or ICU Admission Within 24 Hours From Emergency Department Arrival eTable 3. Performance of EWSs for Predicting Death or ICU Admission Within 24 Hours From Emergency Department Arrival at Discrete PPV Thresholds eTable 4. Calibration Accuracy of EWSs Based on Brier Scores for Predicting 24-Hour Death or ICU Admission Upon Emergency Department Arrival eTable 5. Predictor Importance for Extreme Gradient Boosting Model Predicting Unexpected Death or ICU Admission Within 24 Hours of Emergency Department Arrival in Patients Aged 80 or More Years eTable 6. Predictor Importance for 2 Separate Extreme Gradient Boosting Models Predicting Unexpected Death or ICU Admission Within 24 Hours of Emergency Department Arrival eFigure 1. Violin Plots Showing the Distribution of Risk Score Values Stratified by 24-Hour Outcome eFigure 2. ROC Curve Analysis of the Evaluated EWS eFigure 3. Positive Predictive Value Across EWS Thresholds eFigure 4. SHAP Values eFigure 5. SHAP Summary Plots for Extreme Gradient Boosting Models Predicting Unexpected Death or ICU Admission Within 24 Hours of Emergency Department Arrival in 2 Age Groups [file jamanetwopen-e261532-s001.pdf]

Supplementary Online Content

Covino M, Cacciamani Fanelli PM, Bonadia N, et al. Early warning scores in emergency department patients aged 80 years or older. *JAMA Netw Open*. 2026;9(3):e261532. doi:10.1001/jamanetworkopen.2026.1532

eMethods
eTable 1. Discriminatory Ability of Early Warning Scores in Predicting Death or ICU Admission Within 24 Hours From ED Arrival
eTable 2. Performance of EWSs for Predicting Death or ICU Admission Within 24 Hours From Emergency Department Arrival
eTable 3. Performance of EWSs for Predicting Death or ICU Admission Within 24 Hours From Emergency Department Arrival at Discrete PPV Thresholds
eTable 4. Calibration Accuracy of EWSs Based on Brier Scores for Predicting 24-Hour Death or ICU Admission Upon Emergency Department Arrival
eTable 5. Predictor Importance for Extreme Gradient Boosting Model Predicting Unexpected Death or ICU Admission Within 24 Hours of Emergency Department Arrival in Patients Aged 80 or More Years
eTable 6. Predictor Importance for 2 Separate Extreme Gradient Boosting Models Predicting Unexpected Death or ICU Admission Within 24 Hours of Emergency Department Arrival
eFigure 1. Violin Plots Showing the Distribution of Risk Score Values Stratified by 24-Hour Outcome
eFigure 2. ROC Curve Analysis of the Evaluated EWS
eFigure 3. Positive Predictive Value Across EWS Thresholds
eFigure 4. SHAP Values
eFigure 5. SHAP Summary Plots for Extreme Gradient Boosting Models Predicting Unexpected Death or ICU Admission Within 24 Hours of Emergency Department Arrival in 2 Age Groups

This supplementary material has been provided by the authors to give readers additional information about their work.

© 2026 Covino M et al. *JAMA Network Open*.

eMethods

Score Calculation

The following Early Warning Scores were calculated for each patient: National Early Warning Score (NEWS) [13], NEWS2 [14], Modified Early Warning Score (MEWS) [22], Rapid Emergency Medicine Score (REMS) [19], and International Early Warning Score (IEWS) [23]. For NEWS2, patients were considered at risk of type 2 respiratory failure if they had a confirmed history of chronic obstructive pulmonary disease (COPD).

To ensure statistical robustness and mitigate sparsity within the dataset, scores with fewer than 20 patient observations were combined with adjacent lower score categories. Specifically, the following adjustments were applied: for NEWS, scores of 18, 17, and 16 were merged with 15; for MEWS, scores of 13, 12, 11, and 10 were combined with 9; for REMS, scores from 26 through 20 were consolidated into 19; for NEWS2, scores of 18, 17, and 16 were combined with 15; and for IEWS, scores of 25 and 24 were merged with 23.

Statistical analysis

Continuous variables, EWS scores, AVPU scale, and GCS are reported as medians [interquartile range] and compared using the Mann–Whitney U test. Categorical variables are presented as absolute numbers (percentages) and compared using the Chi-square test or Fisher's exact test, as appropriate.

For patients with fewer than three missing parameters, imputation was performed using Monte Carlo methods [21]; all imputed variables had less than 5% missing data points to ensure that imputation was applied only when the extent of missing data was minimal, improving the reliability of the estimates and minimizing bias introduced by data reconstruction. The imputation model included available physiological parameters, ED triage code, chief complaint, and patient age. Imputed values were constrained within the observed range for each parameter. Due to its non-linear distribution, GCS was imputed using interquartile range limits.

Violin plots were adjusted to visually reflect differences in group sizes. The "deceased/ICU 24h" group was scaled by 0.25 (corresponding to approximately 10 times the frequency of the adverse event) to account for its smaller relative sample size; smaller scaling factors were avoided as they made the visualization of the violin plots difficult to interpret. A fixed bandwidth of 0.46 (mean of the two Scott's bandwidths) was applied to ensure consistent kernel density smoothing across groups.

Score performance was assessed in terms of discrimination and calibration. Discrimination refers to the score's ability to distinguish between patients with and without the outcome, while calibration reflects the agreement between predicted and observed event probabilities.

Score discrimination analysis

Receiver operating characteristic (ROC) curve analysis was used to evaluate discrimination. AUROCs were compared using the DeLong method. PPV thresholds of 5%, 10%, and 25%

© 2026 Covino M et al. *JAMA Network Open*.

were selected. For each threshold, the corresponding EWS value at or above, but closest to, the target PPV was identified. At these EWS cutoffs, the observed PPV (95% CI), NPV (95% CI), sensitivity (95% CI), and specificity (95% CI) were calculated and reported. Confidence intervals were calculated using the Wilson score method. PPV was plotted against each EWS value and compared with the theoretical maximum PPV based on outcome prevalence and perfect discrimination.

Score calibration analysis

Calibration was assessed using Brier scores, with 95% confidence intervals and p-values derived from bootstrap resampling (1,000 iterations with replacement), and results were compared to both a null model and the best-performing score.

Brier score (BS) is the mean squared difference between the predicted probability of an event and the event occurrence

$$BrierScore = 1/Nsum_{i=1}^{N}(p_i - o_i)^2$$

The lower the BS, the better the model is at giving probability predictions that match reality, and the less "off" the probability estimates.

Additionally, calibration plots illustrating observed versus predicted probabilities were generated for each EWS, with the dashed line indicating perfect calibration. Calibration slopes were derived from logistic regression models including each EWS as the sole predictor. Model training and probability estimation were internally validated via stratified 10-fold cross-validation.

Spline analysis

The discriminative performance of Early Warning Scores (EWS) was evaluated across patient age using a spline-based, age-stratified analysis restricted to individuals aged 80 to 100 years. Sliding age windows of five years, advancing in one-year increments, were applied to compute the AUC within each interval. The AUC and corresponding 95% confidence intervals were estimated using bootstrap resampling (n = 250). The association between age and AUC was modeled with restricted cubic splines (RCS) fitted via ordinary least squares regression with three degrees of freedom. Spline regression models were compared with fixed-age (intercept-only) models to determine the statistical significance (F-test) of age effects.

Supervised Machine learning analysis (XGBoost)

To assess the relative contribution of individual score components in the entire cohort, an exploratory Extreme Gradient Boosting (XGBoost) model incorporating core variables from all scores was developed. Contributions were quantified using raw and normalized (divided by the maximum value) Shapley Additive Explanations (SHAP) values. Optimized model hyperparameters were: 500 boosting trees, a learning rate of 0.05, a maximum tree depth of 5, a minimum child weight of 30, and a subsampling rate of 0.8. The random state was fixed at 42 to ensure reproducibility. Model training was optimized using the area under the precision-recall curve (AUCPR) as the primary metric. Model training and evaluation were

© 2026 Covino M et al. *JAMA Network Open*.

performed using 10-fold stratified cross-validation. A threshold of 0.05 was selected to optimally balance sensitivity and positive predictive value, maximizing both detection capability and predictive accuracy. CIs were calculated via the bootstrap method for 1,000 resampled means for the SHAP values, and across-fold metric values for cross-validated metrics (ROC-AUC, Sensitivity, Specificity, NPV, PPV).

Spline analysis was employed to identify the age at which the discriminative performance (AUC) of the NEWS exhibited the greatest variation in its rate of change, corresponding to the second derivative of the spline function. The inflection point was observed at 92 years and was consistent across most other scoring systems. Consequently, the cohort was divided at 86 years, representing the midpoint between 80 years and the age of maximal AUC rate change. This threshold represents the onset of appreciable variation in AUC performance with advancing age. Separate XGBoost analyses, consistent with the previously described modeling framework, were performed to assess differences in the relative contributions of predictors within each age stratum. All modeling procedures, including data preprocessing and hyperparameter settings, were identical across groups to maintain methodological consistency. Differences in SHAP values between age groups were evaluated using two-sided nonparametric bootstrap tests on the difference in normalized absolute SHAP values, as per the following equation:

$$p = 2 * \min[P(\Delta \geqq 0), P(\Delta \leqq 0))]$$
$$\Delta = norm_{over\ 86} - norm_{under\ 86}$$

Data were analyzed using IBM SPSS Statistics for Windows, Version 25 (IBM Corp., Armonk, NY, USA), MedCalc Statistical Software version 19.2.1 (MedCalc Software Ltd., Ostend, Belgium; 2020), and R (Version 4.5.1). Custom programming in Python™ 3.3.13 was used to run XGBoost analysis and calculate SHAP values. A two-sided p-value ≤ 0.05 was considered statistically significant in all the analyses.

© 2026 Covino M et al. *JAMA Network Open*.

eTABLE 1. Discriminatory ability of early warning scores in predicting death or ICU admission within 24 hours from ED arrival.

EWS Score	ROC-AUC (95% CI)	ΔAUC	p-value
NEWS	0.782 (0.767 - 0.798)	0	1
NEWS2	0.776 (0.761 - 0.791)	-0,006	< 0.001
REMS	0.752 (0.736 - 0.768)	-0,03	< 0.001
MEWS	0.747 (0.731 - 0.763)	-0,035	< 0.001
IEWS	0.779 (0.764 - 0.795)	-0,003	0,04

Abbreviations: ROC, Receiver Operating Characteristic; AUC, Area Under the Curve; CI, Confidence Interval
ED, Emergency Department. **Notes:** Statistically significant p-values may occur despite overlapping CIs, as the DeLong test assesses paired difference while accounting for correlation. ΔAUC is calculated with respect to the score with the best discriminatory performance: NEWS.

© 2026 Covino M et al. *JAMA Network Open*.

**eTABLE 2**	Performance of EWSs for predicting death or ICU admission within 24 hours from emergency department arrival.				
**EWS**	**Threshold**	**Sensitivity (95% CIs)**	**Specificity (95% CIs)**	**PPV (95% CIs)**	**NPV (95% CIs)**
NEWS	15	1.38 (0.9-2.2)	99.95 (99.9-100.0)	41.46 (27.8-56.6)	97.60 (97.5-97.7)
	14	2.76 (2.0-3.8)	99.89 (99.9-99.9)	38.20 (28.8-48.6)	97.63 (97.5-97.8)
	13	4.79 (3.7-6.1)	99.71 (99.7-99.8)	29.35 (23.5-36.0)	97.67 (97.5-97.8)
	12	8.11 (6.7-9.8)	99.43 (99.4-99.5)	26.25 (22.1-30.9)	97.75 (97.6-97.9)
	11	11.19 (9.6-13.1)	98.97 (98.9-99.1)	21.26 (18.3-24.6)	97.81 (97.7-97.9)
	10	16.71 (14.7-18.9)	98.07 (97.9-98.2)	17.77 (15.7-20.1)	97.92 (97.8-98.0)
	9	23.68 (21.4-26.1)	96.61 (96.4-96.8)	14.83 (13.3-16.5)	98.07 (97.9-98.2)
	8	31.31 (28.8-33.9)	94.31 (94.1-94.5)	12.06 (11.0-13.2)	98.21 (98.1-98.3)
	7	41.52 (38.8-44.3)	90.78 (90.5-91.0)	10.10 (9.3-11.0)	98.42 (98.3-98.5)
	6	51.34 (48.5-54.1)	85.67 (85.4-86.0)	8.21 (7.6-8.8)	98.60 (98.5-98.7)
	5	61.80 (59.1-64.5)	79.52 (79.2-79.9)	7.00 (6.5-7.5)	98.82 (98.7-98.9)
	4	71.94 (69.4-74.4)	70.06 (69.7-70.5)	5.66 (5.3-6.0)	99.01 (98.9-99.1)
	3	83.86 (81.7-85.8)	57.29 (56.9-57.7)	4.67 (4.4-5.0)	99.30 (99.2-99.4)
	2	88.73 (86.8-90.4)	45.52 (45.1-46.0)	3.91 (3.7-4.1)	99.39 (99.3-99.5)
	1	93.67 (92.2-94.9)	36.06 (35.6-36.5)	3.53 (3.3-3.7)	99.56 (99.5-99.7)
	0	100.00 (99.7-100.0)	0.00 (0.0-0.0)	2.43 (2.3-2.6)	nan (nan-nan)
MEWS	9	1.95 (1.3-2.9)	99.98 (100.0-100.0)	68.57 (52.0-81.4)	97.61 (97.5-97.7)
	8	3.65 (2.7-4.8)	99.90 (99.9-99.9)	47.37 (37.6-57.3)	97.65 (97.5-97.8)
	7	6.24 (5.0-7.7)	99.64 (99.6-99.7)	30.20 (24.9-36.1)	97.71 (97.6-97.8)
	6	12.49 (10.8-14.5)	99.03 (98.9-99.1)	24.37 (21.2-27.9)	97.84 (97.7-98.0)
	5	21.98 (19.8-24.4)	97.32 (97.2-97.5)	16.99 (15.2-18.9)	98.04 (97.9-98.2)
	4	35.44 (32.8-38.2)	92.30 (92.1-92.5)	10.30 (9.4-11.2)	98.28 (98.2-98.4)
	3	54.10 (51.3-56.9)	83.47 (83.1-83.8)	7.55 (7.0-8.1)	98.65 (98.5-98.8)
	2	77.21 (74.8-79.5)	54.55 (54.1-55.0)	4.07 (3.8-4.3)	98.97 (98.8-99.1)
	1	91.81 (90.1-93.2)	33.91 (33.5-34.3)	3.35 (3.2-3.5)	99.40 (99.3-99.5)
	0	100.00 (99.7-100.0)	0.00 (0.0-0.0)	2.43 (2.3-2.6)	nan (nan-nan)
REMS	19	1.87 (1.2-2.8)	100.00 (100.0-100.0)	92.00 (75.0-97.8)	97.61 (97.5-97.7)
	18	2.76 (2.0-3.8)	99.99 (100.0-100.0)	82.93 (68.7-91.5)	97.63 (97.5-97.8)
	17	3.73 (2.8-4.9)	99.97 (99.9-100.0)	74.19 (62.1-83.4)	97.65 (97.5-97.8)
	16	4.95 (3.9-6.3)	99.91 (99.9-99.9)	57.01 (47.5-66.0)	97.68 (97.5-97.8)
	15	7.46 (6.1-9.1)	99.78 (99.7-99.8)	45.77 (39.0-52.7)	97.74 (97.6-97.9)
	14	10.71 (9.1-12.6)	99.55 (99.5-99.6)	37.18 (32.3-42.3)	97.81 (97.7-97.9)
	13	17.03 (15.0-19.2)	99.04 (99.0-99.1)	30.79 (27.4-34.4)	97.95 (97.8-98.1)
	12	24.17 (21.9-26.6)	97.99 (97.9-98.1)	23.12 (20.9-25.5)	98.11 (98.0-98.2)
	11	33.90 (31.3-36.6)	95.27 (95.1-95.5)	15.18 (13.9-16.6)	98.30 (98.2-98.4)
	10	45.42 (42.7-48.2)	89.39 (89.1-89.7)	9.65 (8.9-10.4)	98.50 (98.4-98.6)
	9	57.66 (54.9-60.4)	81.37 (81.0-81.7)	7.17 (6.7-7.7)	98.72 (98.6-98.8)
	8	76.40 (73.9-78.7)	56.20 (55.8-56.6)	4.17 (3.9-4.4)	98.96 (98.8-99.1)
	7	82.81 (80.6-84.8)	51.26 (50.8-51.7)	4.07 (3.8-4.3)	99.17 (99.1-99.3)
	6	100.00 (99.7-100.0)	0.00 (0.0-0.0)	2.43 (2.3-2.6)	nan (nan-nan)
IEWS	23	0.16 (0.0-0.6)	100.00 (100.0-100.0)	100.00 (34.2-100.0)	97.57 (97.4-97.7)
	22	0.89 (0.5-1.6)	99.97 (99.9-100.0)	42.31 (25.5-61.1)	97.59 (97.4-97.7)
	21	2.11 (1.4-3.1)	99.91 (99.9-99.9)	37.14 (26.8-48.9)	97.61 (97.5-97.7)
	20	4.14 (3.2-5.4)	99.77 (99.7-99.8)	30.54 (24.1-37.9)	97.66 (97.5-97.8)

© 2026 Covino M et al. *JAMA Network Open*.

	19	7.06 (5.8-8.6)	99.50 (99.4-99.6)	25.97 (21.6-30.9)	97.72 (97.6-97.8)

eTABLE 2 Performance of EWSs for predicting death or ICU admission within 24 hours from emergency department arrival.

EWS	Threshold	Sensitivity (95% CIs)	Specificity (95% CIs)	PPV (95% CIs)	NPV (95% CIs)
	18	10.14 (8.6-11.9)	99.12 (99.0-99.2)	22.32 (19.1-26.0)	97.79 (97.7-97.9)
	17	14.11 (12.3-16.2)	98.33 (98.2-98.4)	17.43 (15.2-19.9)	97.87 (97.7-98.0)
	16	20.60 (18.4-22.9)	97.12 (97.0-97.3)	15.13 (13.5-16.9)	98.00 (97.9-98.1)
	15	28.55 (26.1-31.1)	95.25 (95.1-95.4)	13.05 (11.8-14.4)	98.16 (98.0-98.3)
	14	37.47 (34.8-40.2)	92.17 (91.9-92.4)	10.67 (9.8-11.6)	98.34 (98.2-98.4)
	13	46.23 (43.5-49.0)	87.79 (87.5-88.1)	8.63 (8.0-9.3)	98.49 (98.4-98.6)
	12	57.18 (54.4-59.9)	82.07 (81.7-82.4)	7.37 (6.9-7.9)	98.71 (98.6-98.8)
	11	68.13 (65.5-70.7)	74.27 (73.9-74.7)	6.20 (5.8-6.6)	98.94 (98.8-99.0)
	10	78.10 (75.7-80.3)	63.51 (63.1-63.9)	5.07 (4.8-5.4)	99.15 (99.0-99.2)
	9	85.97 (83.9-87.8)	51.84 (51.4-52.3)	4.26 (4.0-4.5)	99.33 (99.2-99.4)
	8	91.16 (89.4-92.6)	40.13 (39.7-40.6)	3.66 (3.5-3.9)	99.45 (99.3-99.5)
	7	96.51 (95.3-97.4)	20.49 (20.1-20.8)	2.94 (2.8-3.1)	99.58 (99.4-99.7)
	6	99.84 (99.4-100.0)	2.27 (2.1-2.4)	2.49 (2.4-2.6)	99.82 (99.4-100.0)
	5	100.00 (99.7-100.0)	0.00 (0.0-0.0)	2.43 (2.3-2.6)	nan (nan-nan)
NEWS2	15	0.73 (0.4-1.4)	99.96 (99.9-100.0)	30.00 (16.7-47.9)	97.58 (97.4-97.7)
	14	1.54 (1.0-2.4)	99.90 (99.9-99.9)	27.94 (18.7-39.6)	97.60 (97.5-97.7)
	13	3.33 (2.5-4.5)	99.74 (99.7-99.8)	24.12 (18.3-31.1)	97.64 (97.5-97.8)
	12	6.33 (5.1-7.8)	99.47 (99.4-99.5)	22.94 (18.8-27.7)	97.70 (97.6-97.8)
	11	8.92 (7.5-10.6)	99.01 (98.9-99.1)	18.33 (15.4-21.6)	97.76 (97.6-97.9)
	10	14.03 (12.2-16.1)	98.16 (98.0-98.3)	16.00 (13.9-18.3)	97.86 (97.7-98.0)
	9	20.92 (18.7-23.3)	96.77 (96.6-96.9)	13.91 (12.4-15.6)	98.00 (97.9-98.1)
	8	28.06 (25.6-30.6)	94.48 (94.3-94.7)	11.26 (10.2-12.4)	98.14 (98.0-98.3)
	7	38.61 (35.9-41.4)	90.92 (90.7-91.2)	9.59 (8.8-10.4)	98.34 (98.2-98.5)
	6	48.91 (46.1-51.7)	85.84 (85.5-86.1)	7.93 (7.3-8.6)	98.54 (98.4-98.6)
	5	59.21 (56.4-61.9)	79.42 (79.1-79.8)	6.70 (6.2-7.2)	98.73 (98.6-98.8)
	4	71.21 (68.6-73.7)	69.65 (69.2-70.0)	5.53 (5.2-5.9)	98.98 (98.9-99.1)
	3	84.91 (82.8-86.8)	55.91 (55.5-56.4)	4.59 (4.3-4.9)	99.33 (99.2-99.4)
	2	90.19 (88.4-91.7)	43.76 (43.3-44.2)	3.85 (3.6-4.1)	99.44 (99.3-99.5)
	1	94.65 (93.2-95.8)	32.48 (32.1-32.9)	3.38 (3.2-3.6)	99.59 (99.5-99.7)
	0	100.00 (99.7-100.0)	0.00 (0.0-0.0)	2.43 (2.3-2.6)	nan (nan-nan)

Abbreviations: Early Wanring Score (EWS), Intensive Care Unit (ICU), Positive Predictive Value (PPV), Negative Predictive Value (NPV).

© 2026 Covino M et al. *JAMA Network Open*.

eTABLE 3 Performance of EWSs for predicting death or ICU admission within 24 hours from emergency department arrival at discrete PPV thresholds

Threshold PPV	EWS	Score ≥	% PPV (95% CI)	% NPV (95% CI)	% Sensitivity (95% CI)	% Specificity (95% CI)
**≥5%**	NEWS	4	5.7 (5.3–6.0)	99.0 (98.9–99.1)	71.9 (69.4–74.4)	70.1 (69.7–70.5)
	NEWS2	4	5.5 (5.2–5.9)	99.0 (98.9–99.1)	71.2 (68.6–73.7)	69.6 (69.2–70.0)
	MEWS	3	7.6 (7.0–8.1)	98.6 (98.5–98.8)	54.1 (51.3–56.9)	83.5 (83.1–83.8)
	REMS	9	7.2 (6.7–7.7)	98.7 (98.6–98.8)	57.7 (54.9–60.4)	81.4 (81.0–81.7)
	IEWS	10	5.1 (4.8–5.4)	99.1 (99.0–99.2)	78.1 (75.7–80.3)	63.5 (63.1–63.9)
**≥10%**	NEWS	7	10.1 (9.3–11.0)	98.4 (98.3–98.5)	41.5 (38.8–44.3)	90.8 (90.5–91.0)
	NEWS2	8	11.3 (10.2–12.4)	98.1 (98.0–98.3)	28.1 (25.6–30.6)	94.5 (94.3–94.7)
	MEWS	4	10.3 (9.4–11.2)	98.3 (98.2–98.4)	35.4 (32.8–38.2)	92.3 (92.1–92.5)
	REMS	11	15.2 (13.9–16.6)	98.3 (98.2–98.4)	33.9 (31.3–36.6)	95.3 (95.1–95.5)
	IEWS	14	10.7 (9.8–11.6)	98.3 (98.2–98.4)	37.5 (34.8–40.2)	92.2 (91.9–92.4)
**≥25%**	NEWS	12	26.2 (22.1–30.9)	97.7 (97.6–97.9)	8.1 (6.7–9.8)	99.4 (99.4–99.5)
	NEWS2	14	27.9 (18.7–39.6)	97.6 (97.5–97.7)	1.5 (1.0–2.4)	99.9 (99.9–99.9)
	MEWS	7	30.2 (24.9–36.1)	97.7 (97.6–97.8)	6.2 (5.0–7.7)	99.6 (99.6–99.7)
	REMS	13	30.8 (27.4–34.4)	98.0 (97.8–98.1)	17.0 (15.0–19.2)	99.0 (99.0–99.1)
	IEWS	19	26.0 (21.6–30.9)	97.7 (97.6–97.8)	7.1 (5.8–8.6)	99.5 (99.4–99.6)

Abbreviations: EWS, Early Warning Score; ICU, Intensive Care Unit; PPV, Positive Predictive Value; NPV, Negative Predictive Value.

© 2026 Covino M et al. *JAMA Network Open*.

**eTABLE 4**	Calibration accuracy of EWSs based on Brier scores for predicting 24-hour death or ICU admission upon emergency department arrival.			
**EWS Model**	**Brier Score (CI)**	**p-value vs REMS**	**p-value vs Null**	**Observed Event Rate**
NEWS	0.0227 (0.0215 – 0.0238)	<0.001	<0.001	0.0243
NEWS2	0.0229 (0.0216 – 0.0241)	<0.001	<0.001	
MEWS	0.0226 (0.0214 – 0.0239)	<0.001	<0.001	
REMS	0.0220 (0.0208 – 0.0232)	1	<0.001	
IEWS	0.0227 (0.0217 – 0.0239)	<0.001	<0.001	
Null Model	0.0238 (0.0225 – 0.0250)	<0.001	1	

Abbreviations: EWS, Early Warning Score; ICU, Intensive Care Unit; CI, Confidence Interval. **Notes:** The best model according to Brier scoring is REMS. The Null Model is a non-informative model predicting constantly the observed event rate for each value (0.0243). P-values remain significant despite overlapping confidence intervals since the statistical tests assess paired differences in performance metrics while accounting for their correlation.

© 2026 Covino M et al. *JAMA Network Open*.

eTABLE 5 Predictor importance for Extreme Gradient Boosting (AI) model predicting unexpected death or ICU admission within 24 hours of Emergency department (ED) arrival on patients aged ≥ 80 years.

Predictor	Mean |SHAP| [95% CI]	Normalized |SHAP|
SpO2	0.6103 [0.6068 – 0.6136]	1,000
Suppl. O2	0.3216 [0.3195 – 0.3237]	0,527
Syst. BP	0.2967 [0.2946 – 0.2987]	0,486
HR	0.2648 [0.2628 – 0.2668]	0,434
GCS	0.2607 [0.2581 – 0.2635]	0,427
AVPU Scale	0.1892 [0.1878 – 0.1905]	0,310
Diast. BP	0.1557 [0.1547 – 0.1568]	0,255
MAP	0.1423 [0.1414 – 0.1431]	0,233
Body Temp.	0.1297 [0.1286 – 0.1308]	0,213
Age	0.1287 [0.1277 – 0.1296]	0,211
RR	0.0932 [0.0925 – 0.0940]	0,153
Gender	0.0878 [0.0873 – 0.0884]	0,144
T2RF Risk	0.0273 [0.0269 – 0.0277]	0,045

Abbreviations: AI, Artificial Intelligence; SHAP, SHapley Additive exPlanations; SaO2, Oxygen Saturation; Suppl. O2, Supplementary Oxygen; Syst, Systolic; HR, Heart Rate; GSC, Glasgow Coma Scale; Diast, Diastolic; MAP, Mean Arterial Pressure; Temp, Temperature; RR, Respiratory Rate; T2RF, Type 2 Respiratory Failure. **Notes:** For all predictors value refer to Supplementary Table 3.

© 2026 Covino M et al. *JAMA Network Open*.

eTABLE 6 Predictor importance for two separate Extreme Gradient Boosting (AI) models predicting unexpected death or ICU admission within 24 hours of emergency department (ED) arrival: one model trained and evaluated on patients aged 80 - 86 years, and the other on patients aged > 86 years.

Predictor	Normalized |SHAP| (Age>86)	Normalized |SHAP| (Age≤86)	Δ Normalized SHAP	p-value
Suppl. O2	1	0.4106	0.5894	< 0.001
SpO2	0.9925	1	-0.0075	0.26
Syst. BP	0.7669	0.4498	0.3171	< 0.001
GCS	0.7603	0.3615	0.3987	< 0.001
HR	0.6065	0.4463	0.1602	< 0.001
Age	0.3701	0.1687	0.2014	< 0.001
Diast. BP	0.3212	0.3054	0.0157	< 0.001
MAP	0.3208	0.2751	0.0457	< 0.001
RR	0.2998	0.1981	0.1017	< 0.001
Body Temp.	0.2661	0.2221	0.0441	< 0.001
Gender	0.1106	0.242	-0.1314	< 0.001
T2RF Risk	0.0399	0.0131	0.0268	< 0.001
AVPU Scale	0.038	0.4216	-0.3836	< 0.001

Abbreviations: AI, Artificial Intelligence; SHAP, SHapley Additive exPlanations; SaO2, Oxygen Saturation; Suppl. O2, Supplementary Oxygen; Syst, Systolic; HR, Heart Rate; GSC, Glasgow Coma Scale; Diast, Diastolic; MAP, Mean Arterial Pressure; Temp, Temperature; RR, Respiratory Rate; T2RF, Type 2 Respiratory Failure.

Notes: P-values reflect two-sided bootstrap tests of the difference in normalized absolute SHAP values between age groups. Included predictors were selected for their established clinical relevance in early warning scores (EWS). For all predictors value refer to Supplementary Table 2.

© 2026 Covino M et al. *JAMA Network Open*.

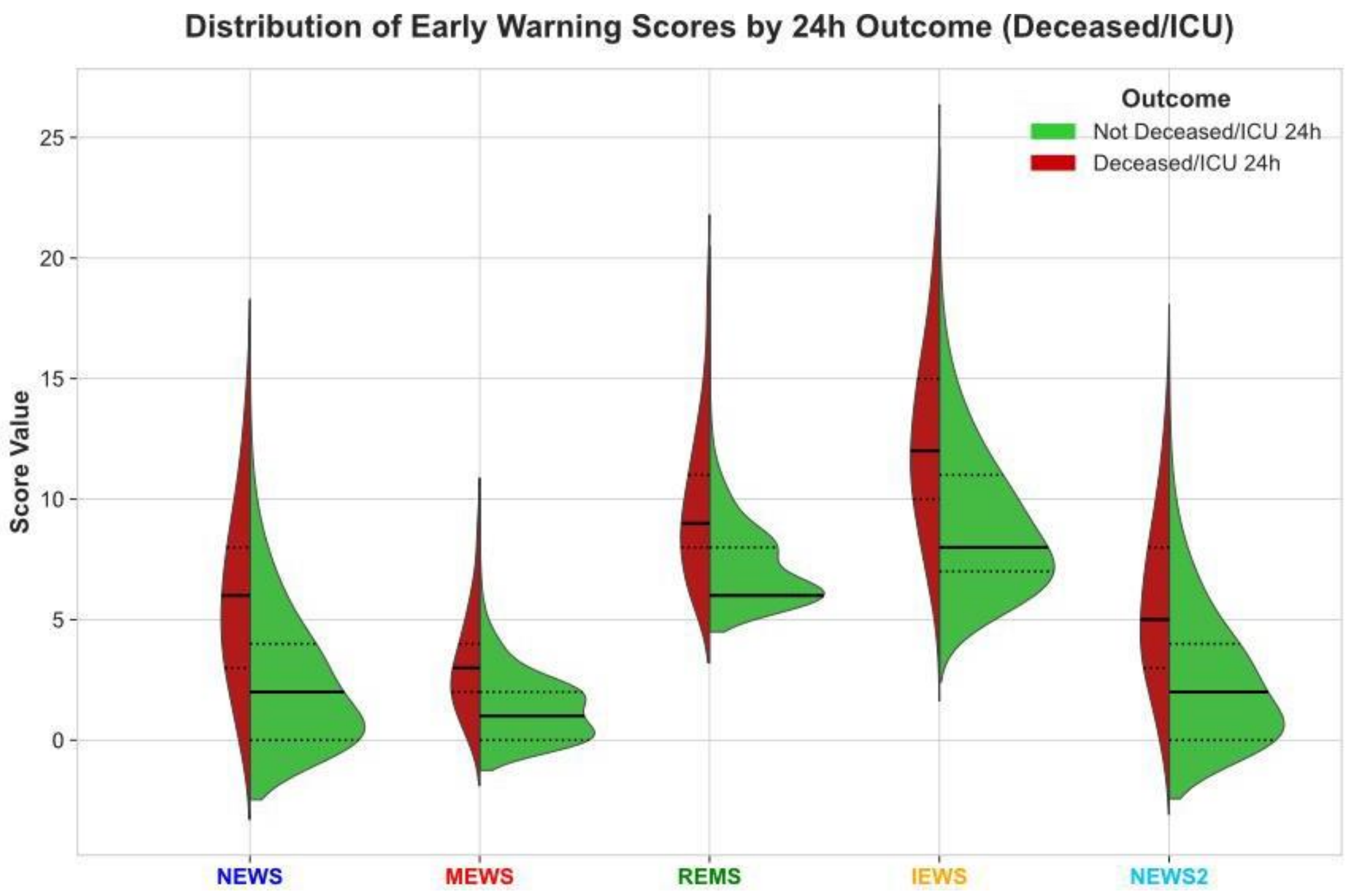

eFigure 1 – Violin plots showing the distribution of risk score values (NEWS, MEWS, REMS, IEWS, NEWS2) stratified by 24-hour outcome (Deceased/ICU vs. Not Deceased/ICU). The solid black lines indicate the median, and the dotted lines represent the first (Q1) and third (Q3) quartiles of the score distributions.

© 2026 Covino M et al. *JAMA Network Open*.

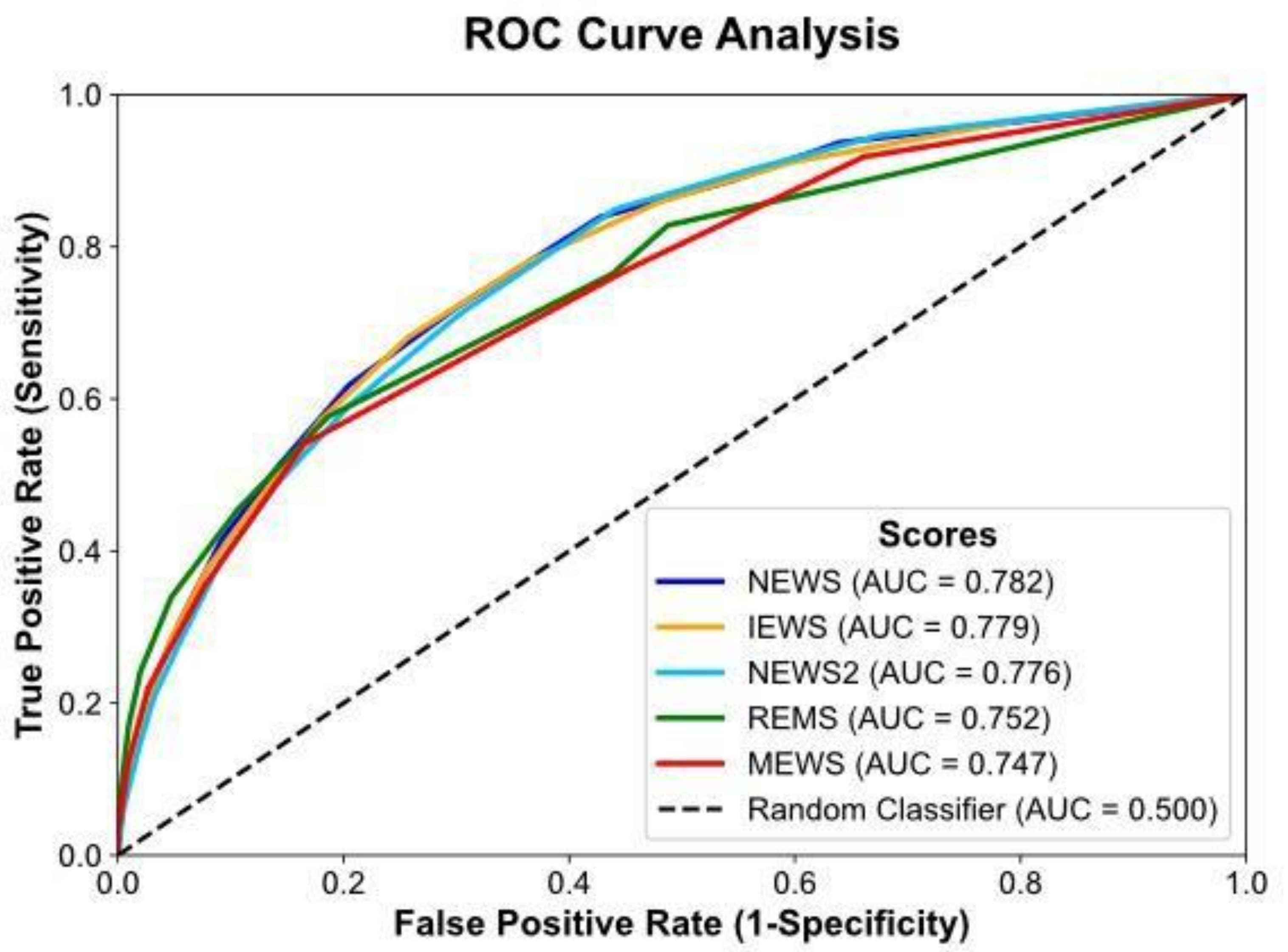

eFigure 2 – ROC Curve analysis of the evaluated EWS. Receiver Operating Characteristics analysis of the evaluated EWS. The ROC AUCs are NEWS 0.782 (0.767 - 0.798), NEWS2 0.776 (0.761 - 0.791), REMS 0.752 (0.736 - 0.768), MEWS 0.747 (0.731 - 0.763), and IEWS 0.779 (0.764 - 0.795).

© 2026 Covino M et al. *JAMA Network Open*.

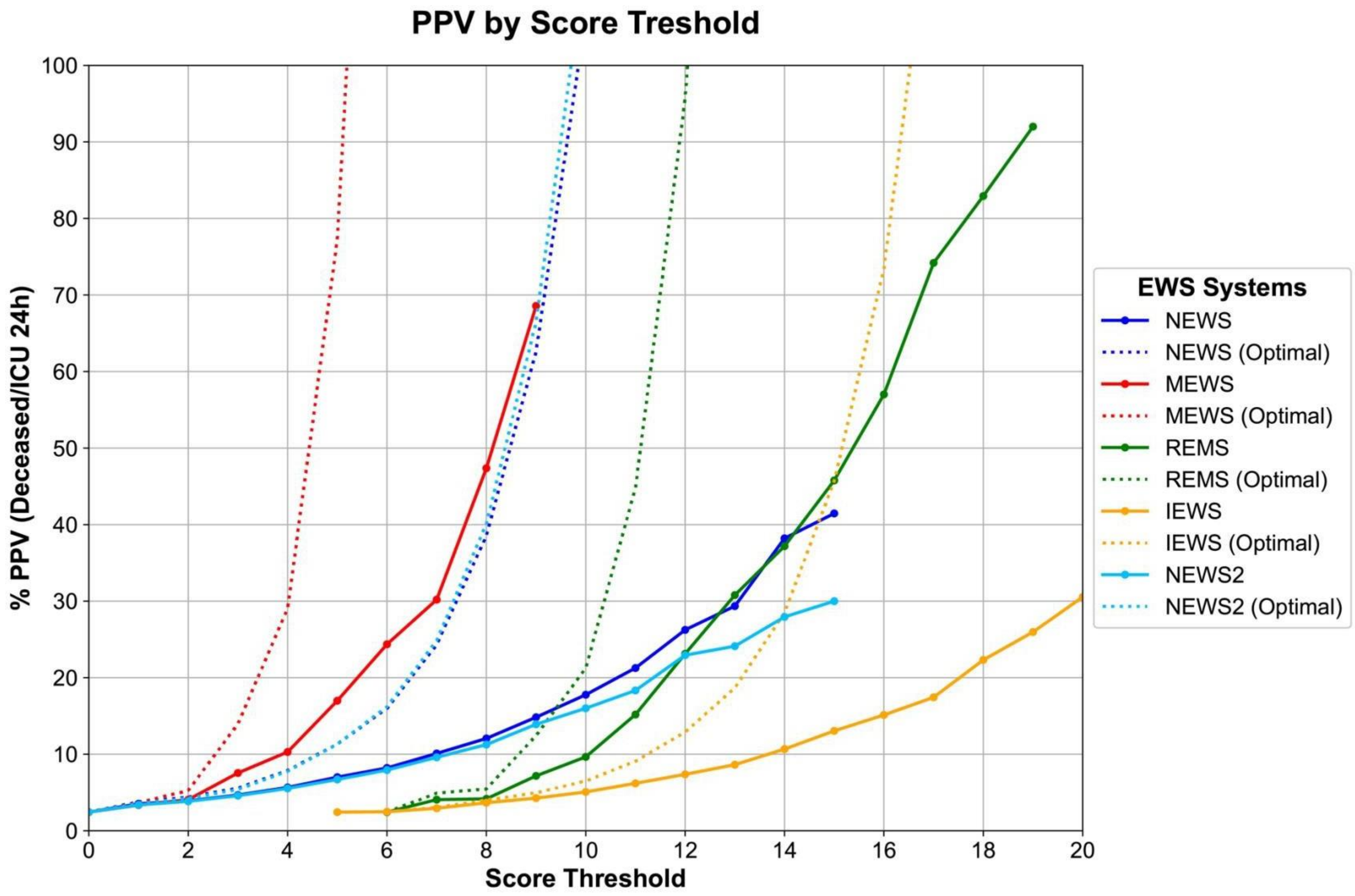

eFigure 3 – Positive predictive value across EWS thresholds. Positive Predictive Value (PPV) for 24-hour Deceased/ICU outcome across score thresholds. Dotted lines show the optimal (theoretical maximum) PPV achievable at each threshold, based on the actual prevalence of the outcome and assuming perfect discrimination (i.e., all true positives are ranked above the threshold). The gap between observed and optimal lines indicates the margin for improvement in each score's ability to concentrate high-risk patients at higher thresholds.

© 2026 Covino M et al. *JAMA Network Open*.

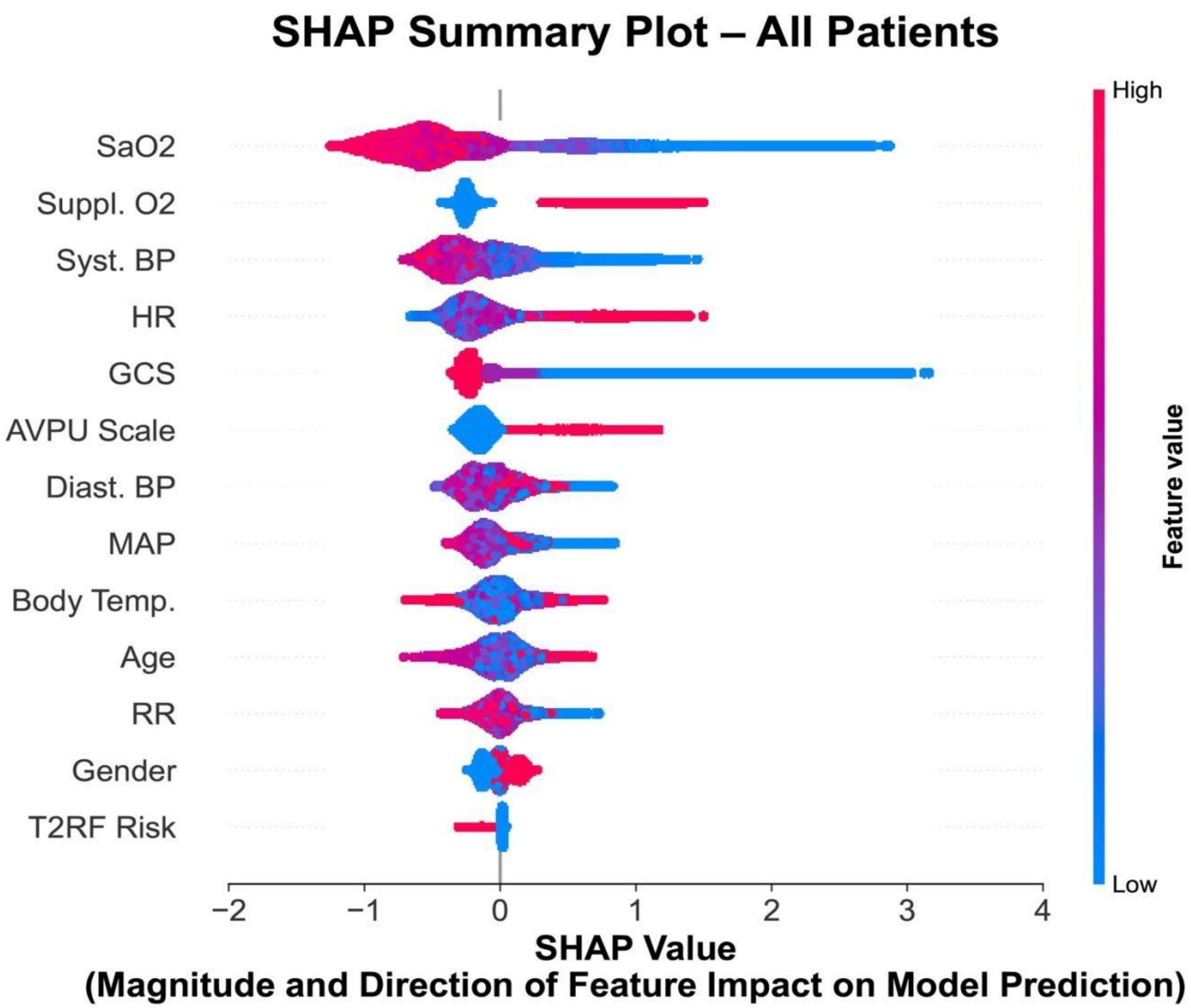

eFigure 4 – SHAP values. The figure represents the Shapley Additive Explanations (SHAP) values for each item included in the evaluated EWS, for the occurrence of death or ICU admission at 24 hours, in the whole study population. The values are derived by a machine learning model based on XGBoost. The direction of the effect and its relevance is plotted in different colours. For instance, lower values of SpO_2 are associated with poor outcome with high impact, whereas only very low or very high temperature show high impact on outcome, both for poor and good outcome.

© 2026 Covino M et al. *JAMA Network Open*.

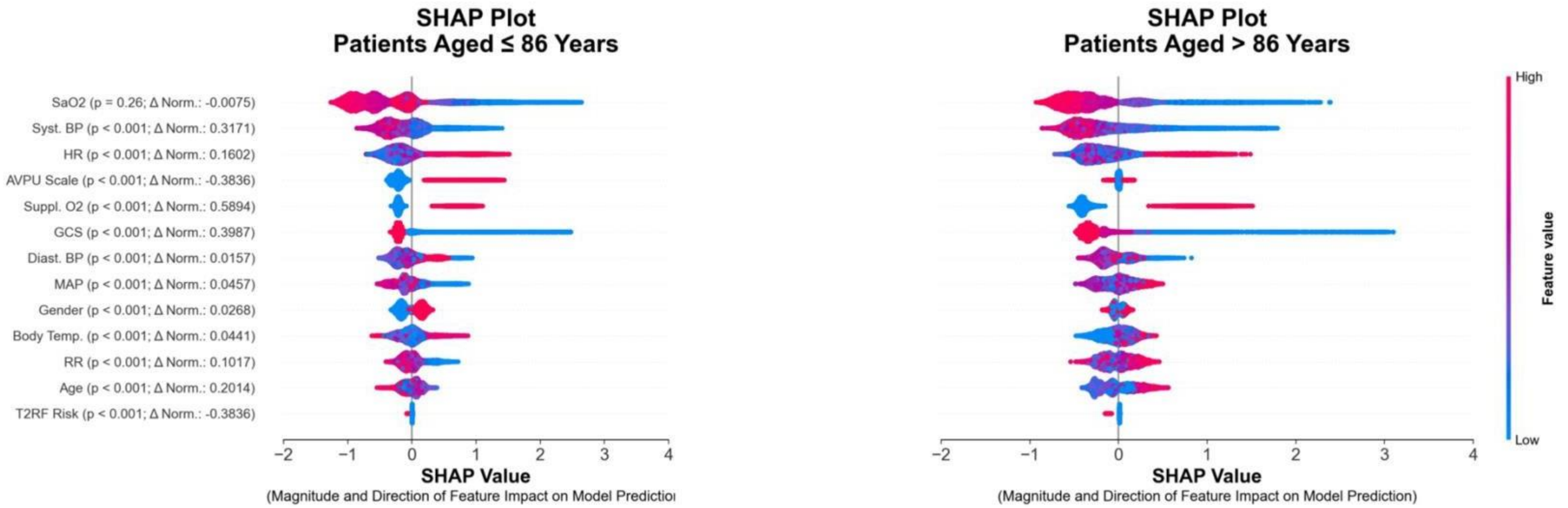

eFigure 5. – SHAP summary plots for Extreme Gradient Boosting models predicting unexpected death or ICU admission within 24 hours of emergency department arrival in two age groups: patients aged > 86 years and patients aged ≤ 86 years. Plotted predictors were selected for their established clinical relevance in early warning scores (EWS). For all predictors value refer to Supplementary Table 2. Each plot shows the magnitude and direction of individual feature contributions to the model predictions, with colors representing feature values (red = high, blue = low). Statistical significance (p-values) of the normalized SHAP values between age groups is reported on the y-axis.

© 2026 Covino M et al. *JAMA Network Open*.
