# Supplementary material for: Early Warning Scores in Emergency Department Patients Aged 80 Years or Older
Source: JAMA Netw Open. 2026 Mar 19;9(3):e261532. doi: 10.1001/jamanetworkopen.2026.1532 (PMC13003372; doi:10.1001/jamanetworkopen.2026.1532)
Supplement: Supplement 2. — Data Sharing Statement [file jamanetwopen-e261532-s002.pdf]

# Data Sharing Statement

Covino. Early Warning Scores in Emergency Department Patients Aged 80 Years or Older. *JAMA Netw Open*. Published March 19, 2026. doi:10.1001/jamanetworkopen.2026.1532

**Data**
**Data available:** Yes
**Data types:** Deidentified participant data
**How to access data:** nicola.bonadia@policlinicogemelli.it
**When available:** With publication

**Supporting Documents**
**Document types:** None

**Additional Information**
**Who can access the data:** Researchers upon reasonable request and for which the use of data is approved by authors
**Types of analyses:** For analysis in the same field
**Mechanisms of data availability:** After approval of a proposal by the authors and by authors' institution and IRB
